# Wnt and Related Signaling Pathways in Melanomagenesis

**DOI:** 10.3390/cancers2021000

**Published:** 2010-05-26

**Authors:** Jesse J. Keller, Randall T. Moon, Andy J. Chien

**Affiliations:** 1 University of Washington School of Medicine, Seattle, WA 98195, USA; E-Mails: jessejkeller@gmail.com (J.J.K.); rtmoon@uw.edu (R.T.M.); 2 The Howard Hughes Medical Institute, 4000 Jones Bridge Road, Chevy Chase, MD 20815-6789, USA; 3 University of Washington Institute for Stem Cell and Regenerative Medicine, Seattle, WA 98109, USA

**Keywords:** Wnts, morphogens, melanoma, signal transduction

## Abstract

Given the pivotal roles of morphogen pathways including Wnt, Notch, Hedgehog, and BMP pathways in the development of the neural crest lineage, it is not surprising that these signaling networks have also been implicated in the biology of malignant melanoma. Understanding the mechanisms by which these pathways can alter cell fate and other biological properties in tumor cells will be essential for determining whether the therapeutic targeting of these pathways has a potential role in melanoma treatment. This review highlights some of the recent findings with regards to how morphogen signaling may regulate melanoma cell biology.

## 1. Introduction

With a doubling time of every 12 years, the incidence of melanoma is increasing at a faster rate than any other cancer [1,2]. Melanoma represents an enormous public health problem, with the American Cancer Society predicting 68,720 new cases of melanoma and 8,650 deaths in 2009. Patients with metastatic disease experience a 5–15% 5-year survival rate that has changed little over the past few decades, despite extensive research efforts. As melanoma has displayed resistance to conventional chemotherapy and radiotherapy, research has focused on unraveling melanoma progression and metastasis so that more effective therapies can be based on understanding the biology of this cancer. 

Compared to other cancers, melanoma has a fairly extensive set of identified signaling pathways and associated mutations implicated in disease progression [3]. These signaling pathways regulate functions essential to tumor cell formation and growth, including immortalization, transformation, proliferation, and migration. In mouse models, targeting specific pathways for melanoma and other cancers has demonstrated enough promise that this approach is receiving intense interest in the clinical setting. To date, efforts to target specific signaling pathways in melanoma patients have been largely disappointing, likely due to factors such as pathway redundancy and de novo mutations that eventually render tumor cells resistant to treatment. In addition, the complex interactions between different signaling pathways in the context of melanoma are not completely understood, making it difficult to accurately predict therapeutic response. 

Morphogen pathways like Wnt, Sonic Hedgehog (Shh), Notch and Bone Morphogenetic Protein (BMP) play an important role in most cancers, including melanoma. Given their ability to regulate cell fate, these pathways have received intense scrutiny as potential leverage to attenuate the metastatic phenotype of melanoma cells [4]. While the concept of a ‘differentiation therapy’ using morphogens is an attractive theory, to date it has not been demonstrated in human patients. The development and use of therapies targeting these important pathways will likely evolve with increased understanding of how signaling pathways interact during melanomagenesis. This review summarizes the major morphogen pathways, and highlights some recent findings relevant for melanoma biology and for future therapeutic development (see also Table 1).

**Table 1:** Studies implicating activated morphogen pathways in melanoma pathogenesis.

Pathway studied	Authors	Pro- or Anti-Melanoma	Context	Notes
Wnt/β-catenin	Chien, *et al*. 2009 [5]	Anti-	Both*	Wnt/β-catenin signaling upregulates differentiation markers, decreases tumor growth and metastasis.
Wnt/β-catenin	Smith *et al*. 2009 [27]	Pro-	*In vitro*	Inhibition of Wnt/β-catenin decreases proliferation
Wnt5A	Dissanayake *et al*. 2008 [18]	Pro-	Both*	Wnt5A activation downregulates antigens important for immune recognition of tumor, increases metastasis
Wnt5A	Witze *et al.* 2008 [31]	Pro-	*In vitro*	Wnt5A signaling enables directional cell movement in response to cues such as chemokine gradients
Wnt5A	Jenei *et al*. 2009 [32]	Pro-	*In vitro*	Direct inhibition of Wnt5A decreases adhesion, invasion, and migration
Wnt5A	O’Connell*et al*. 2009 [37]	Pro-	*In vitro*	Wnt5A activates calpains, cleaving Filamin A, a cytoskeletal protein important in melanoma cell motility
Wnt5A	O’Connell *et al*. 2009 [38]	Pro-	*In vitro*	HSPG’s syndecan-1 and -4 potentiate Wnt5A signaling and increase invasiveness and metastasis
Wnt5A	Schwartz *et al.* 2009 [39]	Pro-	*In vitro*	Constitutive TLR-3 expression is associated with constitutive Wnt5A activity
Wnt5A	O’Connell *et al*. 2010 [36]	Pro-	Both*	ROR2 receptor necessary for Wnt5A-mediated metastasis of melanoma cells
Notch	Bedogni *et al*. 2008 [50]	Pro-	Both*	Oncogene Akt requires Notch to transform melanocytes under hypoxic conditions and increases growth by increased proliferation and decreased apoptosis
Notch	Hu *et al*. 2009 [48]	Anti-	*In vitro*	Notch blockade leads to defective angiogenesis and hypoxia which favors melanoma progression
Notch	Pinnix *et al*. 2009 [46]	Pro-	*In vitro*	N_ICD_ increases proliferation, adhesion, migration, and caused melanocytes to grow at clonal density, proliferate in limited media, and exhibit anchorage-independent growth
Shh	Das *et al*. 2009 [54]	Pro-	Both*	Shh target genes increase proliferation, migration, growth and metastasis of melanoma cells
BMP-2, -4	Rothhammer *et al*. 2008 [56]	Pro-	*In vitro*	BMP-2 and -4 increase expression of matrix metalloproteinases
BMP-7	Na *et al*. 2009 [57]	Anti-	*In vitro*	BMP7 induces mesenchymal to epithelial transition, reduces invasion and migration

* Both = *in vitro* and *in vivo.*

## 2. Discussion

### 2.1. An Overview of Wnt Signaling Pathways

During the course of the last 20 years, much work has been done to elucidate the mechanisms and functions of the Wnt signaling pathway [5]. Research accelerated with the discovery that the *Drosophila* wingless (Wg) gene is the ortholog of the vertebrate *int-1* oncogene [6], which was quickly followed by studies showing the dysregulation of this pathway in numerous cancer models. In the time that followed, developmental model systems such as *Drosophila*, *Xenopus*, and zebrafish have all been instrumental in shedding much needed light on this pathway. As a highly conserved signaling system, the Wnt/Wg pathway holds important roles throughout development from stem cell maintenance and proliferation to differentiation and cell fate specification [5]. Futhermore, the pathway has been implicated in tumor suppression in some contexts, while in other contexts it regulates oncogenesis, adhesion, invasion, and migration [7]. 

Wnts, which form a class of 19 secreted ligands, have been described as acting through two or more distinct signaling mechanisms [5]. Most Wnt isoforms signal through the so-called Wnt/β-catenin pathway, whereas a few isoforms, notably Wnt4, Wnt5A, and Wnt5B signal often through a β-catenin independent, or non-canonical pathway, though depending on context these Wnts can also activate β-catenin [5]. In the absence of Wnt ligand, a “destruction” complex consisting of the three proteins AXIN, glycogen synthase kinase 3-β (GSK3B), and APC (the gene responsible for familial adenomatous polyposi colorectal cancer) constitutively targets the β-catenin protein for proteasomal degradation. In the case of Wnt/β-catenin signaling, binding of Wnt to its cognate receptor(s) inhibits the destruction complex, allowing β-catenin to escape destruction and to accumulate in the nucleus where it enhances transcription of Wnt target genes, most notably through interacting with the T-cell factor/lymphoid enhancer factor (TCF/LEF) family of transcription factors [5]. The non-canonical pathways include one that signals via intracellular calcium (Wnt/Ca pathway) [8] and another that signals through a group of proteins involved in planar cell polarity in *Drosophila* that constitute the “PCP” pathway [9]. It should be noted that Wnt signaling effects are very cell type and tissue context specific, and that these pathways are not mutually exclusive. There is likely cross-talk between the two systems [10], and even antagonism of Wnt/β-catenin signaling by non-canonical pathways [11]. 

### 2.2. Wnt β-catenin Signaling in Melanocytes and Melanoma

Wnt/β-catenin signaling is essential in the formation of melanocytes, a potential cell of origin for malignant melanoma. Melanocytes are derived from neural crest, and various Wnts play a role in the induction, development, and expansion of neural crest as well as the development of melanocytes from neural crest cells [12,13,14,15,16]. It has been postulated that β-catenin dependent Wnt signaling is responsible for the expression of specific melanocytic antigens such as MART1 and SI (gp100) and is important for positioning and differentiation of melanoblasts during development [17]. Meanwhile, β-catenin independent Wnt signaling, mediated via Wnt5A, is capable of dedifferentiating melanocytes and other cells to a more stem cell-like state [18]. This model is also supported by similar findings in hematopoietic stem cells [19]. As the general relationship between development and oncogenesis in several human cancers becomes increasingly stronger, it is not surprising then that the Wnt pathways that play such a critical role in melanocyte development are also implicated in melanomagenesis. 

Although activation of Wnt/β-catenin signaling is thought to play a prominent role in other cancer syndromes such as familial adenomatous polyposis, its role is not as straightforward in melanoma. For instance, activating mutations in APC or β-catenin are relatively rare in melanoma cells [20]. Subsequent studies showed that constitutive activation of the Wnt/ß-catenin pathway was found in only about 30% of melanomas [20]. Later, studies using a transgenic mouse model [21] demonstrated that melanocyte-targeted constitutive overexpression of β-catenin in mice was neither sufficient for increased proliferation of melanocytes nor for conversion to melanoma. This finding was somewhat unexpected given prior reports showing the activation of Wnt/β-catenin signaling led to increased proliferation of cultured melanoma cells through the transcription factor MITF [22]. However, melanocytes were immortalized in culture with constitutive β-catenin activation through downregulation of p16INK4a, a protein which regulates RB1 and thus has an important tumor suppressor function. By down-regulating p16INK4a, constitutively active β-catenin overcomes barriers to senescence in immortalizing melanocytes. However immortalization alone is not sufficient to produce melanoma, and it is only when constitutively active β-catenin is combined with deregulated proliferation through NRAS that full-fledged transformation will occur. Whereas single transgenic mice carrying a melanocyte-specific activating NRAS mutation alone produced few tumors over a long period of time, the overwhelming majority of double transgenic mice (activated β-catenin with activated NRAS) developed melanomas over a shorter span, and many of them developed multiple primary tumors.

Interestingly, several studies suggest that activated Wnt/β-catenin signaling, as measured by nuclear β-catenin in patient melanomas, has a positive correlation with increased patient survival [23,24,25,26]. This finding parallels *in vitro* and *in vivo* results seen with B16 murine melanoma cells overexpressing Wnt3A, an activator of the β-catenin pathway [24]. Implantation of Wnt3A-expressing tumor cells into mice led to decreased tumor growth and metastasis when compared to controls. Additionally, transcriptional profiling revealed that Wnt3A may exert these effects by its ability to upregulate markers of differentiation in melanoma, similar to its function during initial melanocyte development. Taken together these studies suggest that activation of the Wnt/β-catenin pathway may participate in initial tumor formation but that somehow β-catenin signaling must be down-regulated in order for melanoma cells to progress further. While Wnt/β-catenin signaling may be involved in some initial aspects of melanomagenesis, at least based on current mouse models, this same pathway may play a protective role once melanoma is formed by maintaining or altering cell fate to a less aggressive phenotype. In this context, it is unclear how to predict the therapeutic potential of the recent observation that thiazolidinediones, PPAR-γ agonists best known for their roles as oral anti-hyperglycemic agents, decrease melanoma proliferation and promote cellular apoptosis through the direct inhibition of β-catenin transcriptional activity [27]. 

### 2.3. WNT5A and β-catenin-independent Wnt Signaling in Melanoma

While the precise mechanism of β-catenin independent Wnt signaling is less well understood, a great deal of research has focused on its role in melanoma metastasis following the initial discovery by microarray studies that Wnt5A was relatively overexpressed in more aggressive melanomas [28]. It was subsequently shown that Wnt5A can activate protein kinase C (PKC) to induce an epithelial to mesenchymal transition which leads to increased adhesion, invasion, and migration [29,30]. Further studies from Weeraratna and colleagues revealed that Wnt5A can control cell polarity, orientation, and directional movement in melanoma cells [31]. *In vivo* administration of a recombinant Wnt5A protein increased metastasis in a mouse model, further highlighting the potential role of this pathway in late-stage melanoma [18]. Interestingly, transcriptional studies suggest that antagonism of Wnt/β-catenin signaling by Wnt5A may contribute to the downregulation of Wnt/β-catenin signaling observed with melanoma progression [18,24]. More recently, it was shown that direct antagonism of Wnt5A signaling by using a targeted, synthetic peptide inhibitor of the Wnt5A pathway could inhibit Wnt5A-dependent and PKC-dependent increases in melanoma cell migration and invasion [32]. While further studies are warranted to see if peptide antagonists of Wnt signaling can achieve the tissue penetration and targeting necessary to achieve a significant therapeutic effect in patients, these ongoing studies in melanoma provide important mechanistic insight into the molecular events that mediate the regulation of cell motility and the metastatic phenotype by β-catenin independent Wnt signaling.

ROR2, an orphan tyrosine kinase receptor known to mediate Wnt5A signaling [33,34,35], is upregulated in metastatic melanoma cells with high Wnt5A [36]. Studies implicate ROR2 receptor in mediating the effects of Wnt5A via internalization of the ROR2 receptor by PKC-activated clathrin-coated pits, leading to subsequent promotion of metastasis [36]. These studies complemented earlier results showing that in melanoma cells, Wnt5A could regulate cleavage of the cytoskeletal protein filamin A in a manner that was dependent on both ROR2 and calcium [37]. In another study, the same group demonstrated that the heparan sulfate proteoglycans (HSPGs) syndecan-1 and -4 can potentiate Wnt signaling by facilitating ligand binding and/or internalization at the cell surface [38]. After noting that HSPGs are expressed at higher levels in melanoma cell lines of high metastatic potential, HSPGs were cleaved with heparinase III in these cell lines causing Wnt5A to accumulate in culture media concomitant with decreased downstream Wnt5A signaling in cells [38]. Another group found an association between constitutive toll-like receptor 3 (TLR-3) activation and constitutive Wnt5A expression in melanoma cell lines [39]. They used phenylmethimazole, an inhibitor targeting TLR-3, to decrease TLR-3 activity, expression and downstream signaling of Wnt5A both *in vitro* and *in vivo*, providing further evidence that targeting pathways that control Wnt5A may be useful for inhibiting melanoma progression. 

### 2.4. Notch Pathway and Melanoma

The Notch pathway has a hand in controlling proliferation, differentiation, cell fate specification, cell survival [40], angiogenesis [41,42], and immune response [43]. This pathway is activated when a Notch ligand contacts a Notch receptor on a neighboring cell and a series of γ-secretase-related cleavages release the Notch intracellular domain (N_ICD_), which translocates from the cell surface to the nucleus where it associates with the transcription factor recombination signal-binding protein J (RBP-J). This transforms N_ICD_ from transcriptional repressor to transcriptional activator of several genes, most notably those of the HES family of transcription factors. In regards to melanoma, Notch appears to function as an oncogene based on the observation that Notch ligands and receptors are found to be upregulated in melanomas and dysplastic nevi compared to common melanocytic nevi [44]. Additionally, HEY1, a HES-related gene that is a final effector of Notch transcriptional activity, was shown to be upregulated in melanoma cells [45]. Blockade of the Notch pathway via inhibition of γ-secretase activity caused apoptosis of melanoma cells while preserving normal melanocytes [46,47], suggesting that melanoma cells depend on Notch signaling for survival. Notch blockade at the level of RBP-J (a downstream Notch-regulated transcription factor) in four types of cancer (lung carcinoma, hepatocellular carcinoma, osteogenic sarcoma, and melanoma) led to defective angiogenesis as evidenced by increased neovascularization and up-regulated hypoxia-inducible factor 1α (HIF1α), leading to hypoxia [48]. While this hypoxia resulted in slowing of tumor growth in carcinoma of lung and liver and osteogenic sarcoma, it allowed melanoma to grow significantly faster, suggesting that Notch contributes to melanoma progression and that hypoxia is important in this process. 

Overexpression of Notch1 receptor in melanoma cell lines can increase their aggressiveness in a manner that is interestingly dependent on upregulation of β-catenin, highlighting the complexity of the signaling interactions that can occur upon manipulating morphogen pathways [49]. Melanocytes with forced overexpression of N_ICD_ display several characteristics of fully transformed melanocytes including the ability to proliferate at clonal density in limited media, loss of contact inhibition, and anchorage-independent growth, providing further evidence that overactive Notch can contribute to melanocytic transformation to melanoma [46]. 

Pathway cross-regulation by Notch is not limited to morphogen pathways. For example, the melanoma oncogene Akt, which signals through a pathway involving PI3-kinase, requires Notch1 to transform melanocytes under hypoxic conditions and to allow tumor growth *in vivo* by maintaining cells in a proliferative state and protecting them from stress-induced cell death [50]. Understanding the temporal activation of Notch in relation to other signaling networks will be critical for developing therapeutic strategies targeting this pathway in melanoma.

### 2.5. Sonic Hedgehog Pathway and Melanoma

The sonic hedgehog (Shh) pathway is involved in cell fate determination during embryogenesis and in tumorigenesis [51]. Its mechanism is somewhat analogous to the Wnt/β-catenin signaling pathway in that Gli, the downstream transcriptional activator of Shh-responsive genes, is retained in a cytoplasmic destruction complex in the absence of ligand binding. When Shh ligand binds to its receptor Patched (PTCH), Ptch releases Smoothened (SMO), a G-protein coupled-like-receptor that allows Gli to enter the nucleus and activate transcription. Regarding skin tumorigenesis, this pathway is perhaps best known for being upregulated in a majority of basal cell carcinomas [52]. While it has been reported that Shh activation is required for proliferation and survival of human melanoma *in vivo* [53], there is relatively less known about this pathway compared to other morphogens. 

In cultured cell models, Shh activation appears to promote melanoma metastasis [54]. Expression of Shh targets including the Gli1 transcription factor and osteopontin (OPN) increases gradually in melanoma cells of increasing metastatic potential. Gli1 silencing led to decreased OPN expression and decreased proliferation, invasion, and migration of melanoma cells *in vitro* and inhibition of growth and metastasis *in vivo*. Add-back of OPN negated these effects. This experiment suggests that increased Shh activation is partially responsible for the progression of malignant melanoma via Gli1 and OPN and that targeting of this pathway presents a promising therapeutic option.

### 2.6. BMP Pathway and Melanoma

Bone morphogenic proteins (BMPs) are morphogens of the TGF-β superfamily [55]. Their functions during embryogenesis include proliferation, differentiation, cell fate specification, apoptosis, angiogenesis, chemotaxis, and matrix formation. Their signaling mechanism involves BMP ligand binding to a type I and type II serine/threonine kinase receptor which allows phosphorylation of a nuclear effector, SMAD 1,5, or 8 which joins with SMAD4 (co-SMAD) and enters the nucleus to activate transcription of BMP target genes. The pathway is perturbed in a number of human cancers including melanoma. In melanoma, BMPs are upregulated although the functional significance of this finding is complex. It appears that BMPs normally have a tumor suppressive effect, and this holds true in early melanomas. However it appears that late-stage melanomas become resistant to the anti-growth and pro-apoptotic effects of BMPs by upregulation of Noggin, a BMP inhibitor. However, other BMP functions remain intact and the remaining high levels of BMPs drive angiogenesis, migration, matrix remodeling, and decreased immune function—all promoters of melanoma progression.

A recent study showed that BMP-2 and 4 can promote the invasion and migration of melanoma cells by stimulating nearby fibroblasts to increase production of matrix metalloproteinases (MMPs) 1,2,3, and 13 [56]. Use of a general MMP inhibitor was able to reduce invasive potential by about 40%, but inhibition of upstream BMPs using antisense technology or expression of the BMP inhibitor chordin could not block MMP expression, suggesting that factors other than BMPs are also critical for regulating the expression of MMPs.

In several human carcinomas, an epithelial-to-mesenchymal transition is associated with increased metastatic potential as cells transition from a tightly-attached sheet to highly mobile individual cells with increased potential to disseminate. Melanocytes, on the other hand, are derived from mesenchymal neural crest. Thus melanoma cells derived from this lineage are already well-equipped to metastasize. Na *et al.* [57] capitalize upon the finding that BMP-7 reverses TGF-β induced epithelial to mesenchymal transition in the bone marrow [58] and show that human recombinant BMP-7 has the ability to induce mesenchymal-to-epithelial transition in melanoma cells *in vitro*, inhibiting proliferation, migration, and invasion. In addition, BMP-7 decreased resistance to cisplatin in melanoma cell lines [57]. These findings suggest that therapeutic application of BMP-7 may have promise in blocking the metastatic process.

## 3. Conclusions

Studies on the Wnt, Notch, Shh, and BMP pathways have boosted our understanding of the complex roles each play in melanoma progression and at the same time uncovered an abundance of potential targets that can be tested for developing future therapeutic intervention. Clearly, future studies that characterize how these signaling pathways interact will be needed to predict the effects of targeted pathway-directed therapies in melanoma patients. It is still unclear whether studies on morphogen pathways performed in cultured melanoma cell lines provide an accurate reflection of their function in the microenvironment of a human tumor, where factors such as hypoxia, angiogenesis, tumor stroma and immune surveillance play significant roles during melanomagenesis, but cannot be easily replicated in the cell culture environment of a plastic dish. Nevertheless, the growing availability of biologically-active molecules that target these pathways will facilitate efforts to develop rationally-designed therapies that will hopefully translate to improved outcomes for patients with metastatic disease. It is our hope that the discussion here contributes to further research on Wnt and related pathways in melanomagenesis that lead to effective therapies in treating melanoma.

## References

[1] Lens M.B., Dawes M. (2004). Global perspectives of contemporary epidemiological trends of cutaneous malignant melanoma. Br. J. Dermatol..

[2] LaRue L., Luciani F., Kumasaka M., Champeval D., Demirkan N., Bonaventure J., Delmas V. (2009). Bypassing melanocyte senescence by β-catenin: a novel way to promote melanoma. Pathol. Biol. (Paris).

[3] Haluska F., Tsao H., Wu H., Haluska F.S., Lazar A., Goel V. (2006). Genetic alterations in signaling pathways in melanoma. Clin. Cancer Res..

[4] Hendrix M., Seftor E.A., Seftor R.E.B., Kasemeier-Kulesa J., Kulesa P.M., Postovit L.M. (2007). Reprogramming metastatic tumour cells with embryonic microenvironments. Nat. Rev. Cancer.

[5] Chien A.J., Conrad W.H., Moon R.T. (2009). A Wnt survival guide: from flies to human disease. J. Invest. Dermatol..

[6] Rijsewijk F., Schuermann M., Wagenaar E., Parren P., Weigel D., Nusse R. (1987). The Drosophila homolog of the mouse mammary oncogene int-1 is identical to the segment polarity gene wingless. Cell.

[7] O’Connell M.P., Weeraratna A.T. (2009). Hear the Wnt Ror: how melanoma cells adjust to changes in Wnt. Pigment Cell Melanoma Res..

[8] Kohn A.D., Moon R.T. (2005). Wnt and calcium signaling: beta-catenin-independent pathways. Cell Calcium.

[9] Veeman M.T., Axelrod J.D., Moon R.T. (2003). A second canon. Functions and mechanisms of beta-catenin-independent Wnt signaling. Dev. Cell.

[10] Mikels A.J., Nusse R. (2006). Purified Wnt5a protein activates or inhibits beta-catenin-TCF signaling depending on receptor context. PLoS Biol..

[11] Weidinger G., Moon R.T. (2003). When Wnts antagonize Wnts. J. Cell Biol..

[12] Dorsky R.I., Moon R.T., Raible D.W. (1998). Control of neural crest cell fate by the Wnt signaling pathway. Nature.

[13] Labonne C., Bronner-Fraser M. (1998). Induction of patterning of the neural crest: a stem-cell like precursor population. J. Neurobiol..

[14] Dunn K.J., Williams B.O., Li Y., Pavan W.J. (2000). Neural crest-directed gene transfer demonstrates Wnt1 role in melanocyte expansion and differentiation during mouse development. Proc. Natl. Acad. Sci.USA.

[15] Dunn K.J., Brady M., Ochenbauer-Jambor C., Snyder S., Incao A., Pavan W.J. (2005). WNT1 and WNT3a promote expansion of melanocytes through distinct modes of action. Pigment Cell Res..

[16] Sakai D., Wakamatsu Y. (2005). Regulatory mechanisms for neural crest formation. Cells Tissues Organs.

[17] Shibahara S., Takeda K., Yasumoto K., Udono T., Watanabe K., Saito H., Takahashi K. (2001). Microphthalmia-associated transcription factor (MITF): multiplicity in structure, function, and regulation. J. Investig. Dermatol. Symp. Proc..

[18] Dissanayake S.K., Olkhanud P.B., O’Connell M.P., Carter A., French A.D., Camilli T.C., Emeche C.D., Hewitt K.J., Rosenthal D.T., Leotlela P.D., Wade M.S., Yang S.W., Brant L., Nickoloff B.J., Messina J.L., Biragyn A., Hoek K.S., Taub D.D., Longo D.L., Sondak V.K., Hewitt S.M., Weeraratna A.T. (2008). Wnt5A regulates expression of tumor-associated antigens in melanoma via changes in signal transducers and activators of transcription 3 phosphorylation. Cancer Res..

[19] Nemeth M.J., Topol L., Anderson S.M., Yang Y., Bodine D.M. (2007). Wnt5a inhibits canonical Wnt signaling in hematopoietic stem cells and enhances repopulation. Proc. Natl. Acad. Sci. USA.

[20] Rimm D.L., Caca K., Hu G., Harrison F.B., Fearon E.R. (1999). Frequent nuclear/cytoplasmic localization of beta-catenin without exon 3 mutations in malignant melanoma. Am. J. Pathol..

[21] Delmas V., Beermann F., Martinozzi S., Carreira S., Ackermann J., Kumasaka M., Denat L., Goodall J., Luciani F., Viros A., Demirkan N., Bastian B.C., Goding C.R., Larue L. (2007). Beta-catenin induces immortalization of melanocytes by suppressing p16INK4a expression and cooperates with N-Ras in melanoma development. Genes Dev..

[22] Widlund H.R., Horstmann M.A., Price E.R., Cui J., Lessnick S.L., Wu M., He X., Fisher D.E. (2002). Beta-catenin-induced melanoma growth requires the downstream target Microphthalmia-associated transcription factor. J. Cell Biol..

[23] Bachmann I.M., Straume O., Puntervoll H.E., Kalvenes M.B., Akslen L.A. (2005). Importance of P-cadherin, beta-catenin, and Wnt5a/frizzled for progression of melanocytic tumors and prognosis in cutaneous melanoma. Clin. Cancer Res..

[24] Chien A.J., Moore E.C., Lonsdorf A.S., Kulikauskas R.M., Rothberg B.G., Berger A.J., Major M.B., Hwang S.T., Rimm D.L., Moon R.T. (2009). Activated Wnt/beta-catenin signaling in melanoma is associated with decreased proliferation in patient tumors and a murine melanoma model. Proc. Natl. Acad. Sci. USA.

[25] Kageshita T., Hamby C.V., Ishihara T., Matsumoto K., Saida T., Ono T. (2001). Loss of beta-catenin expression associated with disease progression in malignant melanoma. Br. J. Dermatol..

[26] Maelandsmo G.M., Holm R., Nesland J.M., Fodstad Ø., Flørenes V.A. (2003). Reduced beta-catenin expression in the cytoplasm of advanced-stage superficial spreading malignant melanoma. Clin. Cancer Res..

[27] Smith A.G., Beaumont K.A., Smit D.J., Thurber A.E., Cook A.L., Boyle G.M., Parsons P.G., Sturm R.A., Muscat G.E. (2009). PPARgamma agonists attenuate proliferation and modulate Wnt-beta-catenin signaling in melanoma cells. Int. J. Biochem. Cell Biol..

[28] Bittner M., Meltzer P., Chen Y., Jiang Y., Seftor E., Hendrix M., Radmacher M., Simon R., Yakhini Z., Ben-Dor A., Sampas N., Dougherty E., Wang E., Marincola F., Gooden C., Lueders J., Glatfelter A., Pollock P., Carpten J., Gillanders E., Leja D., Dietrich K., Beaudry C., Berens M., Alberts D., Sondak V. (2000). Molecular classification of cutaneous malignant melanoma by gene expression profiling. Nature.

[29] Weeraratna A.T., Jiang Y., Hostetter G., Rosenblatt K., Duray P., Bittner M., Trent J.M. (2002). Wnt5a signaling directly affects cell motility and invasion of metastatic melanoma. Cancer Cell.

[30] Dissanayake S.K., Wade M., Johnson C.E., O’Connell M.P., Leotlela P.D., French A.D., Shah K.V., Leotlela P.D., Hewitt K.J., Rosenthal D.T., Indig F.E., Jiang Y., Nickoloff B.J., Taub D.D., Trent J.M., Moon R.T., Bittner M., Weeraratna A.T. (2007). The Wnt5A/protein kinase C pathway mediates motility in melanoma cells via the inhibition of metastasis suppressors and initiation of an epithelial to mesenchymal transition. J. Biol. Chem..

[31] Witze E.S., Litman E.S., Argast G.M., Moon R.T., Ahn N.G. (2008). Wnt5a control of cell polarity and directional movement by polarized redistribution of adhesion receptors. Science.

[32] Jenei V., Sherwood V., Howlin J., Linnskog R., Säfholm A., Axelsson L., Andersson T. (2009). A t-butyloxycarbonyl-modified Wnt5a-derived hexapeptide functions as a potent antagonist of Wnt5a-dependent melanoma cell invasion. Proc. Natl. Acad. Sci. USA.

[33] Saldanha J., Singh J., Mahadevan D. (1998). Identification of a Frizzled-like cysteine rich domain in the extracellular region of developmental receptor tyrosine kinases. Protein Sci..

[34] Oishi I., Suzuki H., Onishi N., Takada R., Kani S., Ohkawara B., Koshida I., Suzuki K., Yamada G., Schwabe G.C., Mundlos S., Shibuya H., Takada S., Minami Y. (2003). The receptor tyrosine kinase Ror2 is involved in non-canonical Wnt5a/JNK signaling pathway. Genes Cells.

[35] Katoh M. (2005). WNT/PCP signaling pathway and human cancer (review). Oncol. Rep..

[36] O’Connell M.P., Fiori J.L., Xu M., Carter A.D., Frank B.P., Camilli T.C., French A.D., Dissanayake S.K., Indig F.E., Bernier M., Taub D.D., Hewitt S.M., Weeraratna A.T. (2010). The orphan tyrosine kinase receptor, ROR2, mediates Wnt5A signaling in metastatic melanoma. Oncogene.

[37] O’Connell M.P., Fiori J.L., Baugher K.M., Indig F.E., French A.D., Camilli T.C., Frank B.P., Earley R., Hoek K.S., Hasskamp J.H., Elias E.G., Taub D.D., Bernier M., Weeraratna A.T. (2009). Wnt5A activates the calpain-mediated cleavage of filamin A. J. Invest. Dermatol..

[38] O’Connell M.P., Fiori J.L., Kershner E.K., Frank B.P., Indig F.E., Taub D.D., Hoek K.S., Weeraratna A.T. (2009). Heparan sulfate proteoglycan modulation of Wnt5A signal transduction in metastatic melanoma cells. J. Biol. Chem..

[39] Schwartz A.L., Malgor R., Dickerson E., Weeraratna A.T., Slominski A., Wortsman J., Harii N., Kohn A.D., Moon R.T., Schwartz F.L., Goetz D.J., Kohn L.D., McCall K.D. (2009). Phenylmethimazole decreases Toll-like receptor 3 and noncanonical Wnt5a expression in pancreatic cancer and melanoma together with tumor cell growth and migration. Clin. Cancer Res..

[40] Artavanis-Tsakonis S., Rand M.D., Lake R.J. (2004). Notch signaling: cell fate control and signal integration in development. Science.

[41] Hofmann J.J., Iruela-Arispe M.L. (2007). Notch signaling in blood vessels: who is talking to whom about what?. Circ. Res..

[42] Gridley T. (2007). Notch signaling in vascular development and physiology. Development.

[43] Maillard I., Fang T., Pear W.S. (2005). Regulation of lymphoid development, differentiation, and function by the Notch pathway. Annu. Rev. Immunol..

[44] Massi D., Tarantini F., Franchi A., Paglierani M., Di Serio C., Pellerito S., Leoncini G., Cirino G., Geppetii P., Santucci M. (2006). Evidence for differential expression of Notch receptors and their ligands in melanocytic nevi and cutaneous malignant melanoma. Mod. Pathol..

[45] Hoek K., Rimm D.L., Williams K.R., Zhao H., Ariyan S., Lin A., Kluger H.M., Berger A.J., Cheng E., Trombetta E.S., Wu T., Niinobe M., Yoshikawa K., Hannigan G.E., Halaban R. (2004). Expression profiling reveals novel pathways in the transformation of melanocytes to melanoma. Cancer Res..

[46] Pinnix C.C., Lee J.T., Liu Z.J., McDaid R., Balint K., Beverly L.J., Brafford P.A., Xiao M., Himes B., Zabierowski S.E., Yshiro-Ohtani Y., Nathanson K.L., Bengston A., Pollock P.M., Weeraratna A.T., Nickoloff B.J., Pear W.S., Capobianco A.J., Herlyn M. (2009). Active Notch 1 confers a transformed phenotype to primary human melanocytes. Cancer Res..

[47] Qin J.Z., Stennett L., Bacon P., Bodner B., Hendrix M.J., Seftor R.E., Seftor E.A., Margaryan N.V., Pollock P.M., Curtis A., Trent J.M., Bennett F., Miele L., Nickoloff B.J. (2004). p53-independent NOXA induction overcomes apoptotic resistance of malignant melanomas. Mol. Cancer Ther..

[48] Hu X.B., Feng F., Wang Y.C., Wang L., He F., Dou G.R., Liang L., Zhang H.W., Liang Y.M., Han H. (2009). Blockade of notch signaling in tumor-bearing mice may lead to tumor regression, progression, or metastasis, depending on tumor cell types. Neoplasia.

[49] Balint K., Xiao M., Pinnix C.C., Soma A., Veres I., Juhasz I., Brown E.J., Capobianco A.J., Herlyn M., Liu Z.J. (2005). Activation of Notch signaling is required for beta-catenin-mediated human primary melanoma progression. J. Clin. Invest..

[50] Bedogni B , Warneke J.A., Nickoloff B.J., Giaccia A.J., Powell M.B. (2008). Notch1 is an effector of Akt and hypoxia in melanoma development. J. Clin. Invest..

[51] Jacob L., Lum L. (2007). Deconstructing the hedgehog pathway in development and disease. Science.

[52] Dahmane N., Lee J., Robins P., Heller P., Ruiz i Altaba A. (1997). Activation of the transcription factor Gli1 and the Sonic hedgehog signaling pathway in skin tumors. Nature.

[53] Stecca B., Mas C., Clement V., Zbinden M., Correa R., Piguet V., Beermann F., Ruiz I Altaba A. (2007). Melanomas require HEDGEHOG-GLI signaling regulated by interactions between GLI1 and the RAS-MEK/AKT pathways. Proc. Natl. Acad. Sci. USA.

[54] Das S., Harris L., Metge B., Liu S., Riker A.I., Samant R.S., Shevde L.A. (2009). The hedgehog pathway transcription factor GLI1 promotes malignant behavior of cancer cells by up-regulating osteopontin. J. Biol. Chem..

[55] Hsu M.Y., Rovinsky S., Penmatcha S., Herlyn M., Muirhead D. (2005). Bone morphogenetic proteins in melanoma: angel or devil?. Cancer Metastasis Rev..

[56] Rothhammer T., Braig S., Bosserhoff A.K. (2008). Bone morphogenetic proteins induce expression of metalloproteinases in melanoma cells and fibroblasts. Eur. J. Cancer.

[57] Na Y.R., Seok S.H., Kim D.J., Han J.H., Kim T.H., Jung H., Lee B.H., Park J.H. (2009). Bone morphogenetic protein 7 induces mesenchymal-to-epithelial transition in melanoma cells, leading to inhibition of metastasis. Cancer Sci..

[58] Zeisberg M., Hanai J., Sugimoto H., Mammoto T., Charytan D., Strutz F., Kalluri R. (2003). BMP-7 counteracts TGF-beta1-induced epithelial-to-mesenchymal transition and reverses chronic renal injury. Nat. Med..

